# Broncho-Vaxom Attenuates Lipopolysaccharide-Induced Inflammation in a Mouse Model of Acute Lung Injury

**DOI:** 10.3390/ijms25137135

**Published:** 2024-06-28

**Authors:** Min-Seok Woo, Dang Long Cao, Eun-Jin Kim, Yi Yeong Jeong, Dawon Kang

**Affiliations:** 1 Department of Physiology, College of Medicine, Gyeongsang National University, Jinju 52727, Republic of Korea; whitewms@naver.com (M.-S.W.); caodanglong97@gmail.com (D.L.C.); eunjin1981@hanmail.net (E.-J.K.); 2 Institute of Medical Sciences, Gyeongsang National University, Jinju 52727, Republic of Korea; 3 Department of Convergence Medical Science, Gyeongsang National University, Jinju 52727, Republic of Korea; 4 Department of Allergy and Respiratory Medicine, College of Medicine, Gyeongsang National University, Jinju 52727, Republic of Korea; 5 Gyeongsang National University Hospital, Jinju 52727, Republic of Korea

**Keywords:** acute lung injury, Broncho-Vaxom, inflammation, lipopolysaccharide, macrophage

## Abstract

Acute lung injury (ALI) is a condition associated with acute respiratory failure, resulting in significant morbidity and mortality. It involves cellular changes such as disruption of the alveolar–capillary membrane, excessive neutrophil migration, and release of inflammatory mediators. Broncho-Vaxom^®^ (BV), a lyophilized product containing cell membrane components derived from eight bacteria commonly found in the respiratory tract, is known for its potential to reduce viral and bacterial lung infections. However, the specific effect of BV on ALI has not been clearly defined. This study explored the preventive effects of BV and its underlying mechanisms in a lipopolysaccharide (LPS)-induced ALI mouse model. Oral BV (1 mg/kg) gavage was administered one hour before the intratracheal injection of LPS to evaluate its preventive effect on the ALI model. The pre-administration of BV significantly mitigates inflammatory parameters, including the production of inflammatory mediators, macrophage infiltration, and NF-κB activation in lung tissue, and the increase in inflammatory cells in bronchoalveolar lavage fluid (BALF). Moreover, BV (3 μg/mL) pretreatment reduced the expression of M1 macrophage markers, interleukins (IL-1β, IL-6), tumor necrosis factor α, and cyclooxygenase-2, which are activated by LPS, in both mouse alveolar macrophage MH-S cells and human macrophage THP-1 cells. These findings showed that BV exhibits anti-inflammatory effects by suppressing inflammatory mediators through the NF-κB pathway, suggesting its potential to attenuate bronchial and pulmonary inflammation.

## 1. Introduction

Acute lung injury (ALI), which can progress to acute respiratory distress syndrome (ARDS), is characterized by sudden, severe lung inflammation and fluid accumulation, impairing oxygenation [1,2,3]. The pathophysiology of ALI is multifaceted, involving inflammation, oxidative stress, and increased alveolar–capillary barrier permeability. This process includes the release of inflammatory cytokines such as tumor necrosis factor-alpha (TNF-α) and interleukin-1 (IL-1) by lung immune cells, along with the activation of endothelial and epithelial cells and neutrophils [4]. Due to the complex cascade of events leading to significant pulmonary dysfunction and respiratory failure, there is an increasing focus on deciphering the underlying mechanisms of lung injury to develop new therapeutic strategies for the treatment of ALI.

Broncho-Vaxom (BV) is an oral medication designed to prevent and treat recurrent respiratory tract infections. It contains lyophilized bacterial lysates from eight common respiratory pathogens (Haemophilus influenzae, Streptococcus pneumonia, Klebsiella pneumonia, Klebsiella ozaenae, Staphylococcus aureus, Streptococcus pyogenes, Streptococcus sanguinis, and Moraxella catarrhalis) and has been validated as safe and effective for children and adults [5,6]. BV enhances the body’s innate defenses and is particularly effective against recurrent or chronic respiratory infections with extended use [7,8,9,10]. In addition, BV has been shown to enhance immune defenses by promoting the production of salivary and bronchoalveolar IgA, as well as serum IgA and IgG [11]. Beyond these effects, BV possesses immunomodulatory functions; it not only induces the terminal maturation of human dendritic cells, enhancing their T cell-stimulatory capacity [12], but also modulates cytokine levels, leading to an increase in Th1-specific IFN-γ and a decrease in Th2-specific IL-4 [13].

BV serves as a macrophage activator [14], with the oral administration of BV shown to increase macrophage activity in the BALF of patients with chronic bronchitis [15]. Alveolar macrophages within the pulmonary alveoli play a pivotal role in upregulating inflammatory mediators in response to bacterial infections [16,17]. These reports indicate that BV could markedly contribute to regulating the immune response in the airways, potentially through the modulation of macrophages and other immune cells. Numerous studies have confirmed the protective properties of BV against chronic respiratory disorders, underlining its role in immune modulation and inflammatory processes. Given the inflammation-driven pathology of ALI, BV could potentially be utilized as a therapeutic option for ALI. In particular, the effect of BV on macrophage-modulating mechanisms and the pathology of ALI remains to be fully understood. This study explored the effects of BV, with a particular focus on the modulation of macrophages, in a mouse model of ALI and macrophage cell lines.

## 2. Results

### 2.1. BV Pre-Administration Mitigates LPS-Induced Inflammation in BALF

**Figure 1:**
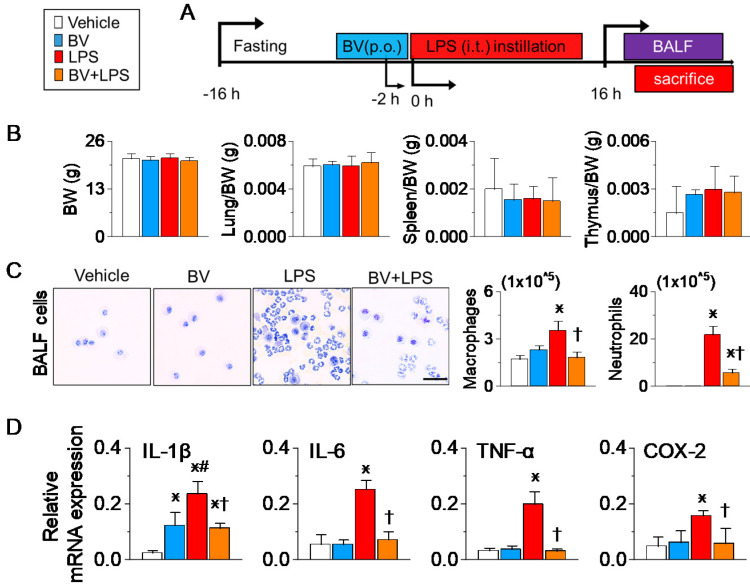
Broncho-Vaxom (BV) pre-administration mitigates LPS-induced inflammation in BALF from an acute lung injury (ALI) mouse model. (**A**) Diagram illustrating the creation of an ALI mouse model. p.o. and i.t. represent oral and intratracheal administration, respectively. (**B**) Comparison of body and organ weights across different experimental groups (each group n = 6). (**C**) Representative Diff-Quick stained images of inflammatory cells in BALF, quantifying macrophages and neutrophils in the accompanying bar graph (each group n = 6). Scale bar, 50 µm. (**D**) Reduction in mRNA levels of pro-inflammatory mediators (IL-1β, IL-6, TNF-α, and COX-2) in macrophages from BALF following BV pre-administration, normalized to GAPDH expression (each group n = 3). The values shown in the bar graph represent mRNA expression levels measured by quantitative PCR. The experiment was run in triplicate. Data are expressed as the mean ± SD. * *p* < 0.05 compared to the vehicle group; ^†^ *p* < 0.05 compared to the LPS group; ^#^ *p* < 0.05 compared to the BV group. The legend box on the left side of A applies to all panels.

Figure 1A illustrates the protocol for assessing the BV effect using an LPS-challenged ALI mouse model. Mice received 1 mg/kg of BV orally two hours before intratracheal LPS administration. Body, lung, spleen, and thymus weights measured 16 h post LPS treatment showed no significant differences across experimental groups (Figure 1B). Total cell counts in BALF were significantly increased in the LPS group compared to the vehicle group (*p* < 0.05), with no differences observed between the vehicle and BV groups (Figure 1C). In addition, BV pretreatment significantly reduced the numbers of macrophages and neutrophils in the BALF of the BV+LPS group (*p* < 0.05, Figure 1C). As shown in Figure 1D, the LPS challenge notably increased the expression of IL-1β, IL-6, TNF-α, and COX-2 in macrophages obtained from BALF compared to the vehicle group. The LPS-induced mRNA expression of these pro-inflammatory mediators was reduced in the BV+LPS group. BV treatment alone did not affect the production of pro-inflammatory mediators, except for IL-1β, where BV groups showed a significant increase in IL-1β mRNA expression levels (*p* < 0.05).

### 2.2. BV Pre-Administration Inhibits LPS-Induced Inflammation in Lung Tissues

**Figure 2:**
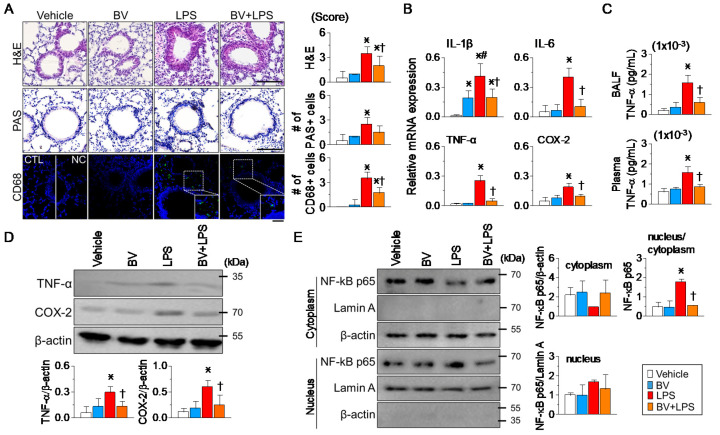
BV mitigates inflammatory responses in lungs from LPS-induced ALI mice. (**A**) Histological analysis shows that BV pre-administration reduces tissue damage and macrophage infiltration in the lungs, as judged by H&E staining, PAS staining, and CD68 immunohistochemistry. NC denotes the negative control without anti-CD68 antibody treatment. DAPI (blue) highlights nuclei, while CD68-positive macrophages are marked with Alexa Fluor 488 (green). Scale bar, 50 µm. Inflammation scores and counts of PAS-positive and CD68-positive cells are quantified in the accompanying bar graphs (each group n = 3). (**B**,**D**) Reductions in inflammatory marker levels in lung tissues post BV treatment, as evidenced by RT-PCR (**B**) and Western blot (**D**) analyses (each group n = 3). (**C**) After BV treatment, decreased TNF-α concentrations in the BALF and plasma of ALI mice (each group n = 6). (**E**) Reduced nuclear translocation of NF-κB p65 in lung tissues after LPS challenge in BV-treated mice (each group n = 3). The β-actin and lamin A were used as markers for the cytoplasm and nucleus, respectively. All experiments were run in triplicate, and the values were summarized. Data are expressed as the mean ± SD. * *p* < 0.05 compared to the control group (vehicle); ^†^ *p* < 0.05 compared to the LPS group; ^#^ *p* < 0.05 compared to the BV group. The legend box at the bottom right side applies to all panels.

The lung histological changes were assessed post LPS challenge, as shown in Figure 2A. H&E staining revealed the typical lung architecture in the vehicle group, while the LPS group displayed signs of immune cell infiltration, hemorrhage, edema, and congestion within the alveolar walls. However, pre-administration of BV notably prevented the severe histopathological changes induced by LPS. PAS staining showed that the pre-administration of BV slightly decreased the number of PAS-positive goblet cells, which had increased in the LPS group. Immunostaining for CD68, a macrophage marker, showed an increased number of green positive cells in the LPS group compared to the vehicle group, with a significant reduction observed in the BV+LPS group (*p* < 0.05, Figure 2A). As shown in Figure 2B, high mRNA expression levels of IL-1β, IL-6, TNF-α, and COX-2 were observed in the LPS group. These elevated expression levels were significantly decreased in the BV+LPS group (*p* < 0.05, Figure 2B). In the BV-alone group, IL-1β mRNA expression levels were significantly increased (*p* < 0.05). Consistent with mRNA levels, TNF-α concentrations in BALF and plasma were significantly lower in the BV+LPS group compared to the LPS group (*p* < 0.05, Figure 2C). Pre-administration of BV significantly downregulated these pro-inflammatory genes (*p* < 0.05). Western blot assays showed that increased TNF-α and COX-2 expression in lung tissue induced by LPS were reduced considerably following BV pre-administration (*p* < 0.05, Figure 2D). Furthermore, the pre-administration of BV significantly inhibited LPS-induced NF-κB nuclear translocation (*p* < 0.05, Figure 2E).

### 2.3. BV Pretreatment Reduces the LPS-Induced Activation of Lung Macrophage MH-S Cells

**Figure 3:**
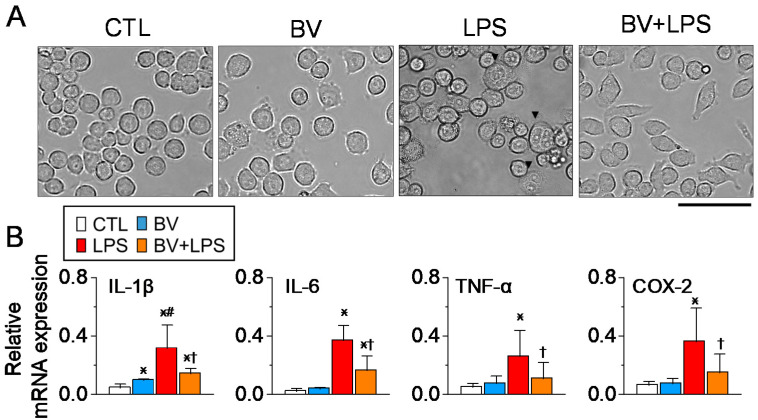
BV pretreatment modulates mRNA expression and morphology in LPS-stimulated MH-S macrophages. (**A**) Morphological changes in mouse alveolar macrophages following LPS stimulation, with and without BV pretreatment, were observed under light microscopy. Arrows in the LPS group indicate cells with notable morphological changes. Scale bar, 50 μm. (**B**) Representative RT-PCR bands showing the effects of BV pretreatment on the expression of specific genes in LPS-stimulated MH-S macrophages. (**B**) Quantitative PCR data with gene expression levels normalized to GAPDH (each group n = 4). All experiments were run in triplicate, and the values were summarized. Data are expressed as the mean ± SD. * *p* < 0.05 compared to the control; ^†^ *p* < 0.05 compared to the LPS treatment; ^#^ *p* < 0.05 compared to the BV group. The legend box at the bottom right side applies to all panels.

To investigate the effect of BV on the LPS-induced activation of lung macrophages, murine alveolar macrophage MH-S cells were pre-treated with BV at a concentration of 3 µg/mL, followed by stimulation with LPS (1 µg/mL). As shown in Figure 3A, compared to the control, LPS-treated cells exhibited characteristics of the M1 phenotype, including a larger, flatter shape with vacuoles. Quantitative PCR analysis showed that LPS treatment increased M1 macrophage markers such as TNF-α, IL-1β, IL-6, and COX-2 (Figure 3B). However, pretreatment with BV mitigated the morphological changes induced by LPS and significantly reduced the expression of these M1 markers (*p* < 0.05). Few cells were visible, exhibiting a large, flat shape with vacuoles in the BV+LPS group. Treatment with BV alone slightly altered the morphology of MH-S cells, but did not affect the expression levels of pro-inflammatory mediators, with the exception of IL-1β.

### 2.4. BV Alleviates the LPS-Induced Activation of Human Macrophages

**Figure 4:**
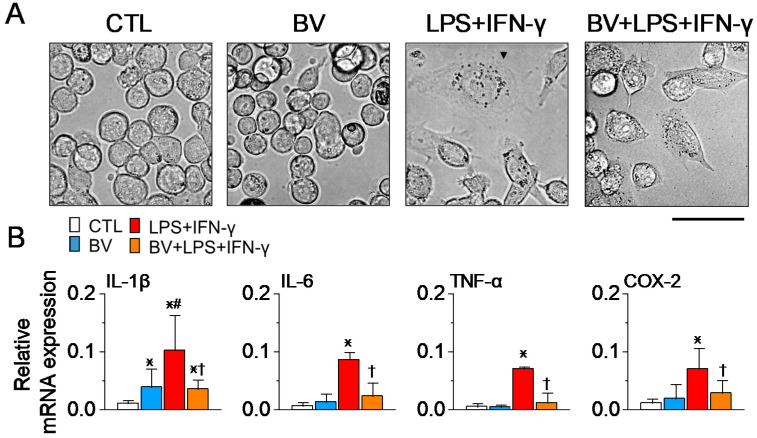
BV pretreatment attenuates M1 macrophage activation in human macrophages exposed to LPS and IFN-γ. (**A**) Morphological changes in PMA-differentiated THP-1 macrophages following exposure to 100 ng/mL LPS and 20 ng/mL IFN-γ for 48 h, with and without BV pretreatment, as observed under light microscopy. The arrow in the LPS+IFN-γ group indicates a cell with notable morphological changes. Scale bar, 50 μm. (**B**) Reduction in mRNA expression levels of pro-inflammatory mediators due to BV pretreatment, as demonstrated by real-time PCR (each group n = 4). All experiments were run in triplicate, and the values were summarized. Data are expressed as the mean ± SD. * *p* < 0.05 compared to the control; ^†^ *p* < 0.05 compared to the LPS treatment; ^#^ *p* < 0.05 compared to the BV group.

PMA-treated THP-1 M0 macrophages were used to assess the effects of BV on the activation of human macrophages. The cells were pre-treated with BV at a concentration of 3 µg/mL for 2 h, followed by stimulation with 100 ng/mL of LPS and 20 ng/mL of IFN-γ for 48 h. Figure 4A shows that no morphological changes were observed in the cells treated only with BV compared to the control. However, pretreatment with BV slightly mitigated the morphological changes (a large, flat shape with vacuoles) typically induced by LPS and IFN-γ (Figure 4A). Moreover, the expression levels of M1 markers (TNF-α, IL-1β, IL-6), which LPS and IFN-γ upregulated, decreased following BV pretreatment (Figure 4B). BV alone increased IL-1β mRNA expression.

## 3. Discussion

This study demonstrates that pretreatment with BV markedly attenuates inflammation in a mouse model of ALI induced by LPS, extending its application from chronic to acute respiratory conditions. Observations include a decrease in inflammatory cell recruitment and pro-inflammatory cytokine levels in BALF and lung tissues without affecting the weights of the body or organs, indicating BV’s targeted action without systemic adverse outcomes. In addition, diminished morphological alterations in M1 macrophages and a decline in inflammatory markers were noted, highlighting BV’s potential role in managing acute respiratory diseases, a field currently limited by the absence of specific therapeutic options.

Upon pathogen exposure, inflammatory mediators, signaling molecules, and transcription factors are activated to combat infection [18,19,20]. Specifically, LPS triggers Toll-like receptor 4 (TLR4), which initiates inflammatory pathways and induces mediators such as inducible nitric oxide synthase and COX-2. This process leads to the release of pro-inflammatory cytokines (TNF-α, IL-1β, IL-6), which are implicated in airway inflammation [18,19,20,21]. Bacterial infections activate the transcription factor NF-κB, which is essential for triggering inflammation-related genes such as cytokines, chemokines, and adhesion molecules [22,23]. Upon activation by the LPS-TLR4 complex, NF-κB undergoes phosphorylation and subsequently translocates into the nucleus. BV pretreatment reduces these signals in BALF and lung tissues exposed to LPS, thereby attenuating LPS-induced lung damage by suppressing critical inflammatory pathways.

Inflammatory airway disorders such as ALI commonly involve immune cell infiltration [24,25,26]. Considering the role of BV in regulating immune cells, administering 2.5 mg of BV diminishes immune cell accumulation and IL-1β secretion in alum-induced peritoneal inflammation, inducing a protective state [27]. Priming alveolar macrophages with BV could improve the overall function of macrophages, accelerating the resolution of inflammation and reducing lung damage [28]. Bacterial lysates, through TLR4 activation, are known to modulate protective antibody responses and enhance macrophage recruitment for a swifter IFN-1 viral response [28,29]. Our results, aligning with previous findings, showed a significant decrease in macrophages and neutrophils in the BALF following BV pretreatment, and decreased levels of pro-inflammatory cytokines (TNF-α, IL-1β, and IL-6) in macrophages. Additionally, we observed a slight increase in macrophage numbers in BALF and lung tissue in the BV group, suggesting the potential to amplify macrophage involvement in subsequent inflammatory responses.

However, in our study, BV alone did not significantly induce pro-inflammatory markers in lung tissues, BALF, or MH-S and THP-1 macrophages, except for an increase in IL-1β. Considering the increased number and altered morphology of macrophages in the BV group, it is likely that IL-1β was involved. Dang et al. reported that BV alone does not activate the inflammasome, but serves as a priming signal for multiple inflammasomes [27]. LPS-treated mice exhibit significantly increased ATP levels [30], which could act as a priming signal for BV to increase IL-1β. Additionally, the internal and external ATP in the cultured cells may have also played a role. In general, bacterial lysates such as BV enhance macrophage recruitment and the secretion of pro-inflammatory mediators [14]. In a study on RAW264.7 cells, BV at 100 μg/mL stimulates the secretion of IL-1β, IL-6, and TNF-α through the activation of TLR4- and TLR2-mediated ERK1/2-NF-κB pathways [31]. Similarly, in human myeloid cells, BV (33~900 μg/mL) interaction with TLR2 and TLR4 elevates the immunoregulatory gene signature, including increased TNF-α, IL-6, and NF-κB activation in a dose-dependent manner [32]. The primary distinction of this study from previous studies lies in the cell types and experimental conditions, particularly the concentration and duration of BV treatment. We believe that the more potent activation of RAW264.7 cells observed could be due to the higher BV concentration and shorter treatment duration than those applied in our study, noting that the macrophage activity tends to decrease over time. Similarly, the study on myeloid cells showed cytokine production and NF-κB activity induction with BV treatments at concentrations exceeding those in our current study.

These findings suggest that the effectiveness of BV might vary with treatment concentration and duration and differ across cell types. BV’s effect could be consistent in inflammatory conditions due to the increased number of activated macrophages, although this might hinge on inflammation severity. In particular, pretreatment with BV will protect against acute inflammation by priming macrophages, thus facilitating the return to normal tissue homeostasis through infection clearance. Consequently, BV represents a novel immunomodulatory strategy, potentially serving as preventive or adjunctive therapy in acute respiratory inflammations. It may block the binding of the SARS-CoV-2 spike protein to human bronchial epithelial cells by altering host–cell membrane proteins and glycosaminoglycans [8], suggesting a clinical role in reducing acute infection-induced exacerbations in those with pre-existing respiratory diseases. Further studies are needed to investigate BV’s optimal timing, dosage, and delivery in acute models to fully understand its therapeutic potential and applicability. In addition, the mechanisms of IL-1β-associated inflammasome regulation by BV need to be investigated.

## 4. Materials and Methods

### 4.1. Broncho-Vaxom Formulation

Broncho-Vaxom^®^ (BV) utilized in this study was supplied by Aju Pharm Co., Ltd. (Seoul, Republic of Korea). The production of BV was a collaborative effort between Aju Pharm and OM Pharma (Geneva, Switzerland). Each BV capsule contains a standardized formulation of lyophilized mixed bacterial lysates, with a concentration of 7 mg per capsule. BV is a lyophilized, fractionated, and alkaline extract derived from eight distinct bacterial strains: Haemophilus influenzae, Streptococcus pneumonia, Klebsiella pneumonia, Klebsiella ozaenae, Staphylococcus aureus, Streptococcus pyogenes, Streptococcus sanguinis, and Moraxella catarrhalis. The process involves lysing the bacterial strains, mixing, filtering, and neutralizing the soluble fractions. Subsequently, the bacterial cell extracts are freeze-dried. For experimental use, the freeze-dried extract within each capsule was reconstituted in distilled water to create a stock solution with a 0.7 mg/mL concentration. This solution was further diluted as required in culture medium for in vitro experiments or distilled water for mouse administration. The vehicle group received a solution of equivalent concentration devoid of active BV components.

### 4.2. Animal Care

Male C57BL/6J mice, six weeks old, were obtained from the Koatech Animal Breeding Center (Pyeongtaek, Republic of Korea). The mice were housed in a pathogen-free environment with unrestricted access to food and water for one week under a 12 h light/dark cycle before the commencement of the experiments. All animal experiments were conducted under the National Institutes of Health Guidelines for the care and use of laboratory animals; the protocol received approval from the Gyeongsang National University Animal Care and Use Committee (GNU-220519-M0049) on 19 May 2022.

### 4.3. Establishment of an ALI Mouse Model

The mice used in the experiment were randomly divided into four distinct groups, each comprising six individuals: group 1 received solvent treatment (vehicle), group 2 was administered with BV only (BV), group 3 was administered with lipopolysaccharide only (LPS), and group 4 was administered with BV and LPS (BV+LPS). The methodology for inducing and treating ALI (group 4) is illustrated in Figure 1. The mice received an oral BV gavage at a 1 mg/kg dosage. Two hours following the BV administration, LPS (2.5 mg/kg, Sigma, St. Louis, MO, USA) was administered via intratracheal injection. BV and LPS were administered to mice that had been fasted overnight to standardize their metabolic state before the experiment. Sixteen hours post-treatment, the mice were anesthetized to facilitate the isolation of bronchoalveolar lavage fluid (BALF). Subsequently, the animals were euthanized, and the lungs, spleen, and thymus were harvested and weighed. The collected samples were stored at −80 °C or fixed in a 4% paraformaldehyde solution for subsequent experiments.

### 4.4. BALF Cell Differential

BALF collection and differential cell counts were performed as per a previously established protocol (Ryu et al., 2022). Briefly, mice were euthanized using tribromoethanol (Sigma), and BALF cells were collected from each mouse lung lavage with 2 mL of cold PBS. BALF cells were centrifuged (300× *g*, 10 min, 4 °C), the supernatant was saved, and the pellet was resuspended in 1 mL of PBS. The cells were then counted using the Countess™ II Automated Cell Counter (AMQAX1000, Life technologies Corp., Bothell, WA, USA). The remaining cells (200 µL) were differentially counted on glass slides prepared by cytocentrifugation (150× *g*, 10 min) and stained with Diff-Quick (Sysmex International Reagents, Kobe, Japan). Slides were processed through sequential staining (Solution A for 10 s, five dips in Solution C, then B) and dehydration (distilled water, 100% ethanol) before being cleared in xylene and mounted. Under a microscope (BX51-DSU; Olympus, Tokyo, Japan), 300 cells per slide were differentiated based on staining patterns, with neutrophils and macrophages identified by specific colorations. BALF samples were preserved at −80 °C for subsequent cytokine analysis.

### 4.5. Real-Time PCR

Total RNA was isolated from mouse BALF cells, macrophage cell lines, and lung tissues using TRIzol^®^ Reagent (Invitrogen, Carlsbad, CA, USA), according to the manufacturer’s protocol. First-strand cDNA was synthesized from 3 µg of total RNA using the DiaStart^TM^ RT kit (SolGent, Daejeon, Republic of Korea). Real-time PCR was conducted using the TOPreal^TM^ SYBR Green 2x PreMix (Enzynomics, Daejeon, Republic of Korea) on a LightCycler^®^ 480 II/96 (Roche Diagnostics Ltd., Rotkreuz, Switzerland). The protocol included an initial denaturation at 95 °C for 5 min, followed by 45 cycles of denaturation at 95 °C for 30 s, annealing at 60℃ for 30 s, and extension at 72 °C for 30 s. Real-time PCR data were analyzed statistically with the 2^−ΔΔCt^ method to determine mRNA level changes, with the mRNA expression of target genes normalized to GAPDH levels (Table 1).

### 4.6. Hematoxylin and Eosin (H&E) and Periodic Acid–Schiff (PAS) Staining

The staining procedures and subsequent analyses followed the protocols described in a previous study (Ryu et al., 2022). Briefly, lung tissue sections were prepared for staining H&E (Sigma) and PAS (Millipore, Billerica, MA, USA). Lung tissues were fixed in 4% paraformaldehyde and embedded in paraffin. After sectioning and deparaffinization, tissues were stained, dehydrated in ascending alcohol grades, cleared in xylene, and mounted. The stained tissues were photographed with a BX61VS microscope (Olympus, Tokyo, Japan).

### 4.7. Immunohistochemistry

Immunohistochemical staining followed the protocols from Ryu et al., 2022. Briefly, lung tissue sections were deparaffinized, permeabilized with 0.2% Triton X-100, and blocked with normal goat serum. They were incubated with anti-CD68 primary antibody (1:100 dilutions, Santa Cruz Biotechnology, Dallas, TX, USA), followed by Alexa Fluor 488-conjugated goat anti-rabbit IgG secondary antibody (1:300, Thermo Fisher Scientific/Invitrogen, Eugene, OR, USA), and stained with 4′,6-diamidino-2-phenylindole (DAPI, Invitrogen). Stained sections were mounted and examined using a confocal laser scanning microscope (Olympus).

### 4.8. Western Blot Analysis

We conducted Western blotting as per our previous protocol [21]. Briefly, proteins were isolated from mouse lung tissues using RIPA buffer (Thermo Fisher Scientific, Rockford, IL, USA) with protease inhibitors (Roche Diagnostics, Indianapolis, IN, USA) and separated by centrifugation. Nuclear and cytoplasmic fractions were obtained through differential centrifugation and lysed accordingly. Protein concentrations were determined using the Pierce bicinchoninic acid protein assay kit (Thermo Fisher Scientific). Equal amounts of protein were loaded on SDS-polyacrylamide gels, electrophoresed, and transferred to polyvinylidene difluoride membranes (Millipore). The membranes were blocked and incubated overnight with primary antibodies against cyclooxygenase-2 (COX-2, #4842S, 1:1000 dilution, Cell signaling, Danvers, MA, USA), TNF-α (#ab6671, 1:1000 dilution, Abcam, Cambridge, UK), NF-κB p65 (#8242S, 1:1000 dilution, Cell Signaling), lamin A (#ab8980, 1:1000 dilution, Abcam), and β-actin (#A5441, 1:5000 dilution, Sigma). Following secondary anti-rabbit or anti-mouse antibody (1:5000 dilution, Assay Designs, Ann Arbor, MI, USA) incubation, signals were detected using enhanced chemiluminescence (Thermo Fisher Scientific) and imaged with an iBright^TM^ CL1500 system (Thermo Scientific Fisher/Life Technologies Holdings Pte Ltd., Marsiling/Woodlands, Singapore). Protein levels were normalized to β-actin or lamin A.

### 4.9. Enzyme-Linked Immunosorbent Assay

TNF-α levels in plasma and BALF were quantified using a mouse TNF-α ELISA kit (R&D Systems, Minneapolis, MN, USA). Following the manufacturer’s protocol, 50 µL of standards or samples were added to designated wells, followed by an antibody cocktail. After an hour’s incubation at room temperature and three washes, 100 µL of substrate solution was added to each well. Following a 15 min incubation, 100 µL of stop solution was introduced, and the wells were mixed. Optical densities were measured within 10 min at 450 nm using a VERSAmax^TM^ microplate reader (Molecular Devices, Sunnyvale, CA, USA).

### 4.10. Cell Culture

The MH-S alveolar macrophage and THP-1 human leukemia monocytic cell lines were obtained from the American Type Culture Collection (ATCC, Manassas, VA, USA). They were cultured in Dulbecco’s modified Eagle’s medium (DMEM, Gibco/Life Technologies, Grand Island, NY, USA) and Roswell Park Memorial Institute (RPMI) 1640 medium, respectively, both supplemented with 10% fetal bovine serum (FBS) and 1% penicillin/streptomycin (all from Gibco/Life Technologies). The cells were cultured at 37 °C in an atmosphere of 95% air and 5% CO_2_, with medium changes every other day. The cultured THP-1 cells were induced to a macrophage-like phenotype with 150 nM phorbol myristate acetate (PMA) for 24 h, then polarized to M1 using 100 ng/mL LPS and 20 ng/mL interferon (IFN)-γ for 48 h. BV at 3 μg/mL was treated two hours before the LPS and IFN-γ treatment.

### 4.11. Data Analysis and Statistics

The bands from agarose gels and Western blots were quantified using ImageJ software (version 1.51, National Institutes of Health, Bethesda, MD, USA). Following the normality test, one-way ANOVA followed by the Bonferroni post hoc test was utilized to analyze group differences using OriginPro2020 (OriginLab Corp., Northampton, MA, USA). Data are presented as the mean ± standard deviation (SD), and the criterion for statistical significance was set at *p* < 0.05.

## 5. Conclusions

In this study, we have demonstrated the potential of BV in mitigating inflammatory parameters in a mouse model of ALI. Notably, pretreatment with BV significantly decreased M1 macrophage polarization, indicating a reduction in acute inflammatory responses. These findings suggest that BV may play a beneficial role in modulating the immune response, presenting a promising avenue for therapeutic intervention in inflammatory conditions. Further research is warranted to explore BV’s full potential, immunomodulation mechanisms, and applicability in clinical settings.

## Figures and Tables

**Table 1:** Primer sequences used for quantitative PCR.

Gene Name	GenBank Accession No.		Primer Sequence (5′-3′)	Size (bp)	Species
*Il1b*	NM_008361.4	Sense	AGAATCTATACCTGTCCTGTGT	139	Mouse
		Antisense	TCCACTTTGCTCTTGACTTCT		
	NM_000576.3	Sense	TACGAATCTCCGACCACCAC	229	Human
		Antisense	AGCGTGCAGTTCAGTGATC		
*Il6*	NM_031168	Sense	ATACCACTCCCAACAGACCT	142	Mouse
		Antisense	TCTGCAAGTGCATCATCGTT		
	NM_000600.5	Sense	ACAGCCACTCACCTCTTCAG	231	Human
		Antisense	TCCAAAAGACCAGTGATGATT		
*Tnf*	NM_013693.3	Sense	ATGGGTTGTACCTTGTCTACT	133	Mouse
		Antisense	TTGACGGCAGAGAGGAGGTT		
	NM_000594.4	Sense	ACCTCTCTCTAATCAGCCCTC	280	Human
		Antisense	ATGCGGCTGATGGTGTGG		
*COX2*	NM_011198.4	Sense	AGTCATTCACCAGACAGATTG	197	Mouse
		Antisense	TGCAGCCATTTCCTTCTCTCC		
	M90100.1	Sence	TGAATGGGGTGATGAGCAGT	137	Human
		Antisense	AAAGTAGTTCTGGGTCAAAT		
*Gapdh*	NM_017008.4	Sense	TGTCATCAACGGGAAGC	166	Mouse
		Antisense	GGAGATGATGACCCGTTT		
	NM_002046.7	Sense	GAACATCATCCCTGCCTCTA	181	Human
		Antisense	CCTGCTTCACCACCTTCTTG		

## Data Availability

The data supporting this study’s findings are available on reasonable request from the corresponding author (D.K.). The article includes all relevant data supporting this study’s conclusions.
